# Comparison of Inner Ear Volume Between Humans and Sheep Using MRI

**DOI:** 10.1007/s10162-025-01002-2

**Published:** 2025-07-18

**Authors:** Fabrice Micaletti, Victoire Simier, Damien Fouan, Jean-Philippe Cottier, J. John Galvin, Jean-Michel Escoffre, David Bakhos

**Affiliations:** 1 ENT Department, University Hospital Center of Tours, 2 Boulevard Tonnellé, 37044 Tours, France; 2 Faculty of Medicine, University of Tours, 10 Boulevard Tonnellé, 37044 Tours, France; 3 Université de Tours, INSERM, Imaging Brain & Neuropsychiatry iBraiN U1253, 37032 Tours, France; 4 Neuroradiology Department, University Hospital Center of Tours, 2 Boulevard Tonnellé, 37044 Tours, France; 5 House Institute Foundation, 2100 W 3rd Street, Suite 111, Los Angeles, CA 90057 USA

**Keywords:** Inner ear, MRI, Large animal, Sheep, Human

## Abstract

**Purpose:**

In preclinical research, animals are used to perform clinical experiments. The use of large animals with human-like anatomies and structural size appears to be essential. For auditory function research, we needed to identify an animal model whose dimensions are close to those of the human inner ear for future research. In the present study, we investigated measurements of the human and sheep inner ear using 3 T Magnetic Resonance Imaging (MRI) to evaluate the suitability of a sheep model for studying the inner ear.

**Methods:**

Inner ears were compared between 8 ears from 4 normal humans (women) and 8 ears from 4 normal sheep (female). Cranial MRI of both species’ cochleae were acquired and analyzed, with specific measurements for key anatomical features, including the cochlea length and width, the length and width of the inner auditory canal, the number of spiral turns of the cochlea and the cochlea volume. The size ratios between sheep and human cochlear structures were calculated and compared.

**Results:**

Overall cochlear dimensions of the sheep were approximately 2/3 that of human cochleae across most measurements, except for the internal auditory canal. The internal auditory canal of the sheep was 1/3 of the size of that in humans. The number of spiral turns in the cochlea was equivalent between the two species.

**Conclusion:**

Given the proportionally similar dimensions to humans, the sheep cochlea appears to be a promising model for inner ear research, specifically to develop pathological models, to study the pathophysiological mechanisms of inner ear diseases, and/or to improve treatment with implantable prostheses.

**Supplementary Information:**

The online version contains supplementary material available at 10.1007/s10162-025-01002-2.

## Introduction

The inner ear (IE) is a complex structure located in the temporal bone that plays a crucial role in hearing and balance. The IE is composed of 2 main parts: the anterior labyrinth, which contains the cochlea—a 2.5-turn spiral organ responsible for converting sound vibrations into neural signals—and the posterior labyrinth, which includes the vestibule and is comprised of three semicircular canals (lateral, anterior and posterior) as well as two otolith organs (utricle and saccule) that are essential for maintaining balance [1, 2]. The IE’s membranous labyrinth contains two distinct fluids—endolymph and perilymph—whose balance is vital; disruptions to this balance can result in conditions such as vertigo, hearing loss and tinnitus [3]. Perilymph fills the scala vestibuli and the scala tympani of the cochlea, while endolymph fills in the scala media [1]. The study of endolymphatic fluids is of crucial importance to understand IE pathologies and developing new therapeutic strategies [4].

However, research is severely hampered by the complex anatomy and sensitive physiology of IE, especially when extrapolating results from animal models to humans. Rodents (guinea pigs, mice and rats) are the most commonly used models, but have IE structures that differ substantially in size, shape, and composition from those of humans. For example, the ratio of round window surface between guinea pigs and humans is estimated to 0.47 (1.18 mm^2^ *vs* 2.98 mm^2^) [5, 6], and the volume of the scala tympani in guinea pigs was estimated at 4.76 μL, compared with 29.22 μL in humans (*i.e.,* ratio 0.16) [7]. The cochlea of the guinea pig has 3.5 turns [8] compared to 2.5 turns in humans. The relevance of rodent-based research to human clinical scenarios is restricted by these anatomical and physiological differences. Developing large animal models (*e.g.,* sheep) for IE research may offer significant advantages over traditional rodent models. The anatomical and physiological similarities between sheep and humans may make sheep an ideal model for studying auditory and vestibular functions, developing surgical techniques, and testing therapeutic interventions, such as implantable prostheses [9]. The structure of the round window membrane in the sheep is similar to humans, with three layers and a thickness between 55 and 71 μm, compared to 70 μm in humans [10]. Similarly, the auditory spectrum is comparable between humans (20–20,000 Hz) and sheep (100–30,000 Hz) [11]. As such, sheep may be a relevant large animal model for hearing research if the dimensions of the cochlea and the volume of endochlear fluids are comparable to those in humans.

By leveraging advanced imaging modalities such as Magnetic Resonance Imaging (MRI), researchers can achieve non-invasive and detailed visualization of the IE's intricate structures, including the compartments that house endolymphatic and perilymphatic fluids. Indeed, MRI allows for accurate measurement and comparison of fluid volumes, shedding light on the similarities and potential functional differences between the IE of sheep and humans. The present study aimed to investigate the IE volumes in both species to further evaluate the suitability of sheep as a large animal model for IE research.

## Material and Methods

### Cohort

**Fig. 1:**
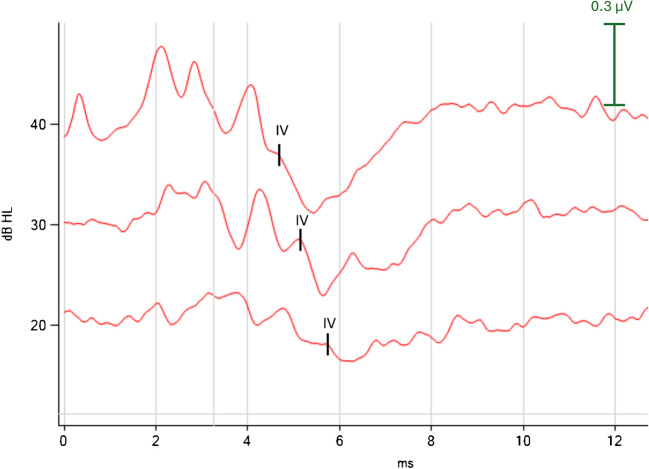
A representative ABR result in sheep for a right ear. Wave IV in sheep is analogous to wave V in human ABR, where it precedes a significant negative deflection. As in humans, an increase in latency is observed with decreasing intensity (dB). When present, wave IV is between 4 and 6 ms. Its presence helps define hearing-norm status

MRI scans were analyzed to compare the anatomical morphology of IEs from 4 normal female humans (mean age = 65.37 ± 13.00 years) and 4 normal female sheep (mean age = 3.57 ± 0.30 years). Only female MRIs were selected to control for potential anatomical differences based on sex assigned at birth, and to ensure consistency with the female sheep cohort. The sheep (Ile de France breed) were sourced from the Unité Expérimentale de Physiologie Animale de l'Orfrasière. The animals were sedated using Isoflurane 3% with oxygen, followed by intravenous ketamine (Ketamidor, Axience SAS, Pantin, France) at 10 mg/kg, and xylazine (Rompun 2%, Bayer, Leverkusen, Germany) at 0.05 mg/kg, and then placed on artificial ventilation. Bone conduction Auditory Brainstem Response (ABR) testing confirmed normal hearing status (NavPRO ONE Bio-logic®, Otometrics, Natus Medical Inc., Middleton, WI, USA) (Fig. 1).

Imaging acquisitions were conducted using a 3 T MRI (Siemens Magnetom Verio syngo MR B19) at the PIXANIM platform, INRAe of Nouzilly. The animal study was reviewed and approved by the Animal Care and Regional Committee for Ethics in Animal Experiments, Centre Val-de-Loire (APAFIS #202,311,170,910,114).

Human MRIs were conducted on patients undergoing standard pre-implantation exploration for an intra-cochlear device, using a 3 T MRI. Experienced radiologists ensured the absence of IE malformations, brain parenchymal tumors, or retro-cochlear anomalies. The clinical study complied with the guidelines of the Declaration of Helsinki.

### MRI Data and Process

**Fig. 2:**
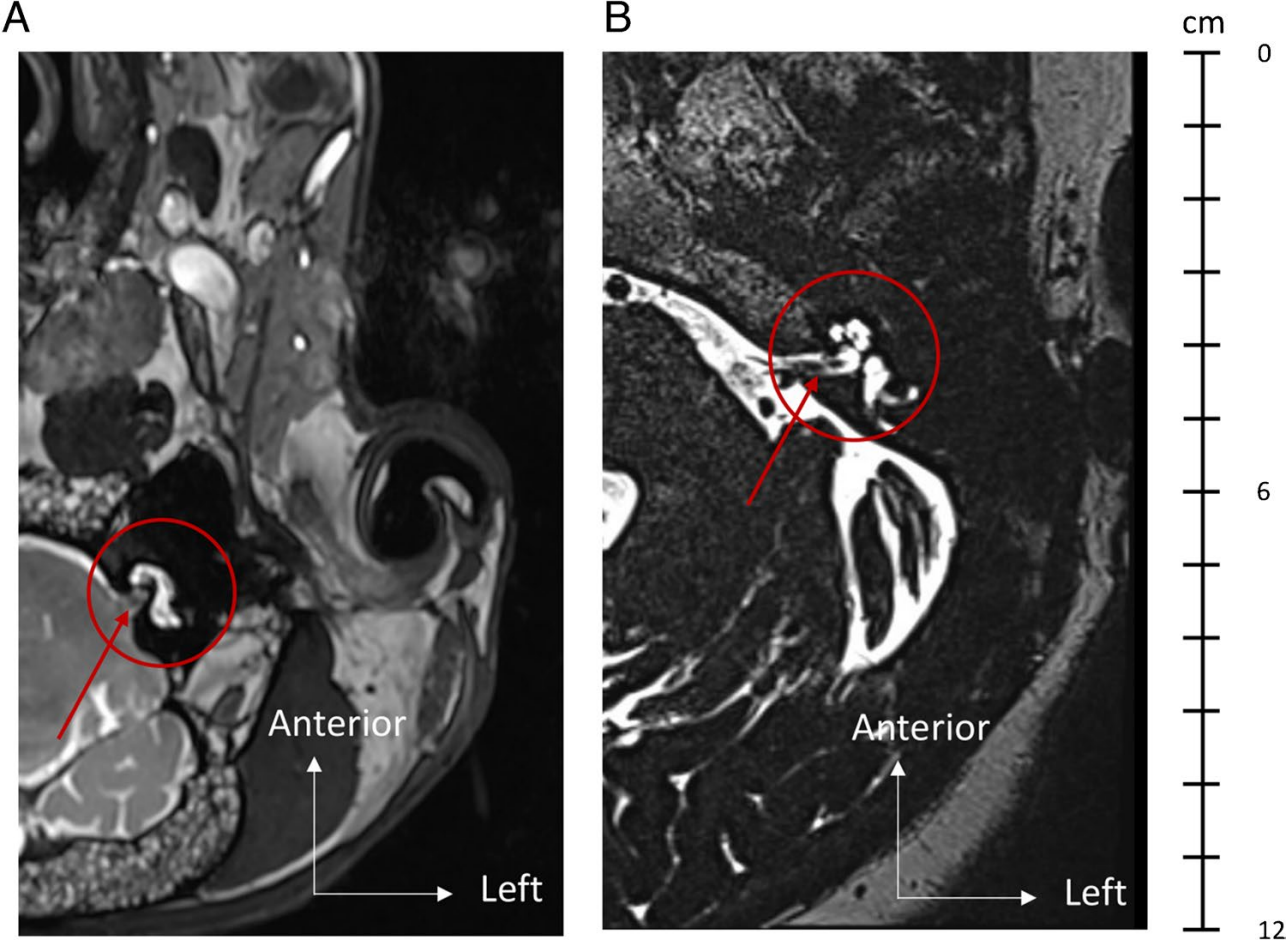
T2 3D CISS (Constructive Interference in Steady State) MRI, in the axial plane, of the left IE of a sheep (**A**) and the left IE of a human (**B**) at the same scale. In this anatomical sequence, the cerebrospinal fluid and IE fluids appear in hypersignal. The red circle shows the IE with the cochlea in front and the vestibule behind. The red arrow indicates the internal auditory canal

A T2 3D Constructive Interference in Steady State (CISS) sequence in the axial plane was used to analyze the images of both sheep and humans (Fig. 2). This high-resolution gradient echo imaging technique is highly sensitive to fluid signals, allowing for clear delineation of the perilymph and endolymph compartments within the cochlea and vestibular system. Scanning parameters were identical for both humans and sheep, with a repetition time (TR) of 8.88 ms, an effective echo time (TE) of 3.94 ms, and a matrix size of 384 × 384 pixels. The voxel size was 0.5 mm × 0.4 mm × 0.4 mm, and the total scanning time was approximately 10 min.

### Building Human and Sheep Models

After retrieving the MRIs in standard DICOM format, the open-source medical imaging analysis software 3D Slicer^®^ (developed at Brigham and Women's Hospital, Boston, Massachusetts, USA) was used for image processing. The images were assessed for the size of specific structures through multiplanar reconstruction, enabling measurements in axial, sagittal, and coronal planes. Using these measurements, 3D Slicer^®^ facilitated segmentation and the calculation of perilymph fluid volumes within the cochlea and vestibule. The CISS sequence from each MRI was imported into the software for image processing. Intensity settings were adjusted to clearly visualize the cochlea and semicircular canals, followed by segmentation of the IE. These settings were tailored to each MRI to achieve optimal thresholding for the semicircular canals while maintaining high IE resolution.

**Fig. 3:**
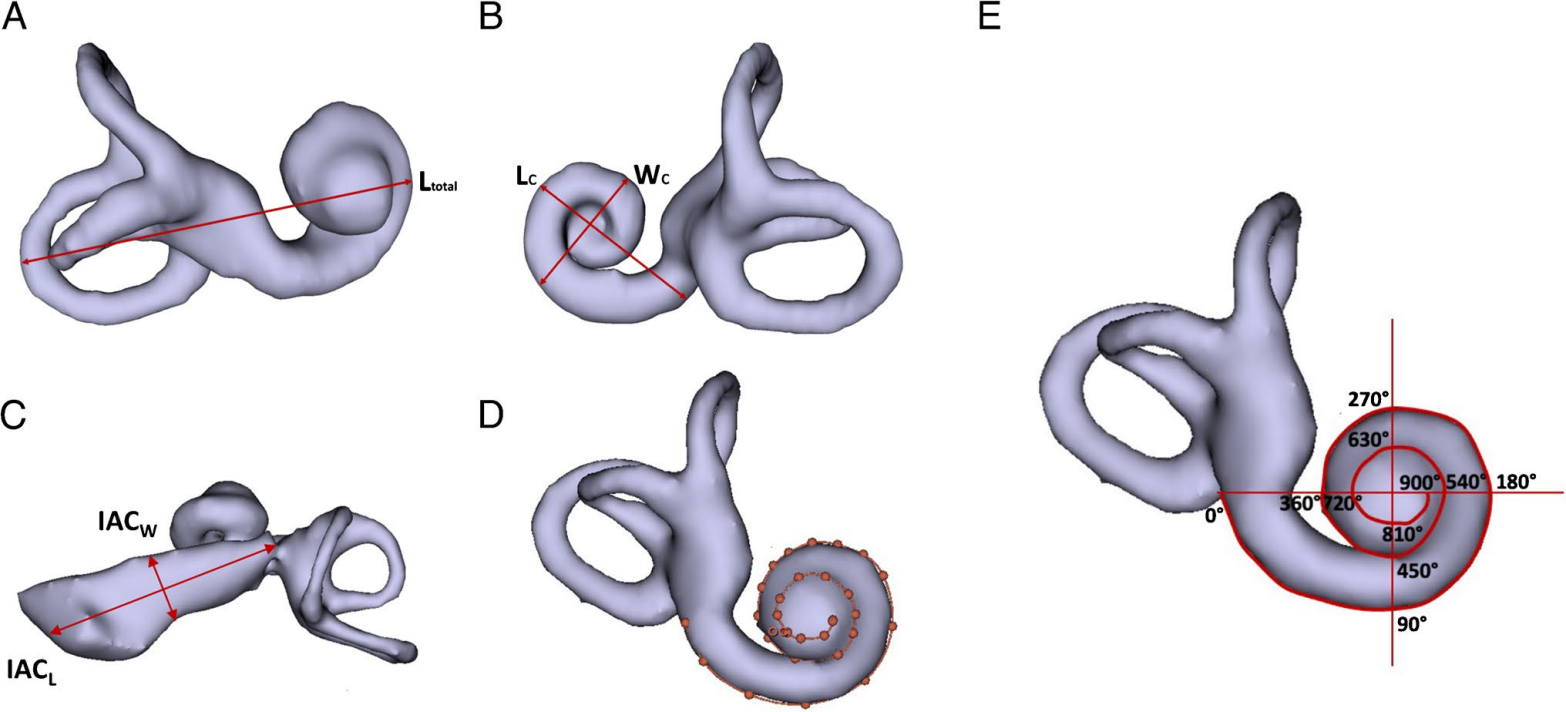
MRI measurements of the inner ear after segmentation on 3D Slicer® software, **A** Inner ear length total (L_total_) along its longest axis, passing through the modiolus; **B** Dimensions of the cochlea with the length (C_L_) passing through the center of the round window and the modiolus and its width (C_W_), perpendicular to the C_L_; **C** Dimensions of the internal auditory canal with length (IAC_L_) and width (IAC_W_); **D** Length of the scala tympani; **E** Number of turns of the cochlea

Following segmentation, the IE was rendered in 3D to enable various measurements using the software’s ruler function in 3D Slicer®, as shown in Fig. 3. The various volumes and measurements carried out were:

i) Inner ear length total (L_total_): in the direction of the greatest length of the IE, line starting from the most posterior point of the posterior semicircular canal to the most anterior point of the basal turn of the cochlea and passing through the central modiolus (Fig. 3A);
ii) Cochlea length (C_L_): in the oblique coronal plane, line starting from the center of the round window membrane (RWM) passing through the central modiolus to the other end of basal turn (Fig. 3B);
iii) Cochlea width (C_W_): in the oblique coronal plane, line perpendicular to the C_L_ value passing through the central modiolus (Fig. 3B);
iv) Length of the inner auditory canal (IAC_L_): In the axial plane, line measuring from the center of the fundus (top end) to the center of the bottom end of the inner auditory canal (IAC) (Fig. 3C);
v) Width of the IAC (IAC_W_): In the axial plane, line measuring perpendicular to the AC_L_ along its center (Fig. 3C);
vi) Scala tympani length: distance obtained with the curve function, from the RWM to the cochlear apex, following the external part of the cochlea (Fig. 3D);
vii) Spiral turns of the cochlea: a horizontal line is drawn at the RWM and passing through the central modiolus with its perpendicular defined. The starting point was set at 0° at the RWM. Each time the line was crossed, 90° was added. One full spiral turn of the cochlea corresponds to 360°, 2 turns to 720° and 2.5 turns to 900°. The spiral turn stops at the most distal point of the RWM, corresponding to the point at the top of the cochlea corresponding to the uppermost point of the cochlea as it follows the outer contour toward the apex (Fig. 3E).

Additionally, the cochlea volume was calculated. This rigorous methodology enables a detailed comparison of IE fluid properties between sheep and humans, using 3 T MRI modalities to provide accurate and comparable information.

### Statistical Analysis

Statistical analyses were performed using GraphPad Prism software version 10.4.0 (GraphPad Software, Inc., La Jolla, CA). Continuous variables were expressed as means and standard deviations. The data followed a non-parametric distribution. Mann–Whitney tests were used to compare the means of continuous variables. For each dimension, a ratio was calculated between the mean sheep and human values. The tests were carried out with a confidence interval of 95%. The significance level was set at *p* < 0.05.

## Results

**Fig. 4:**
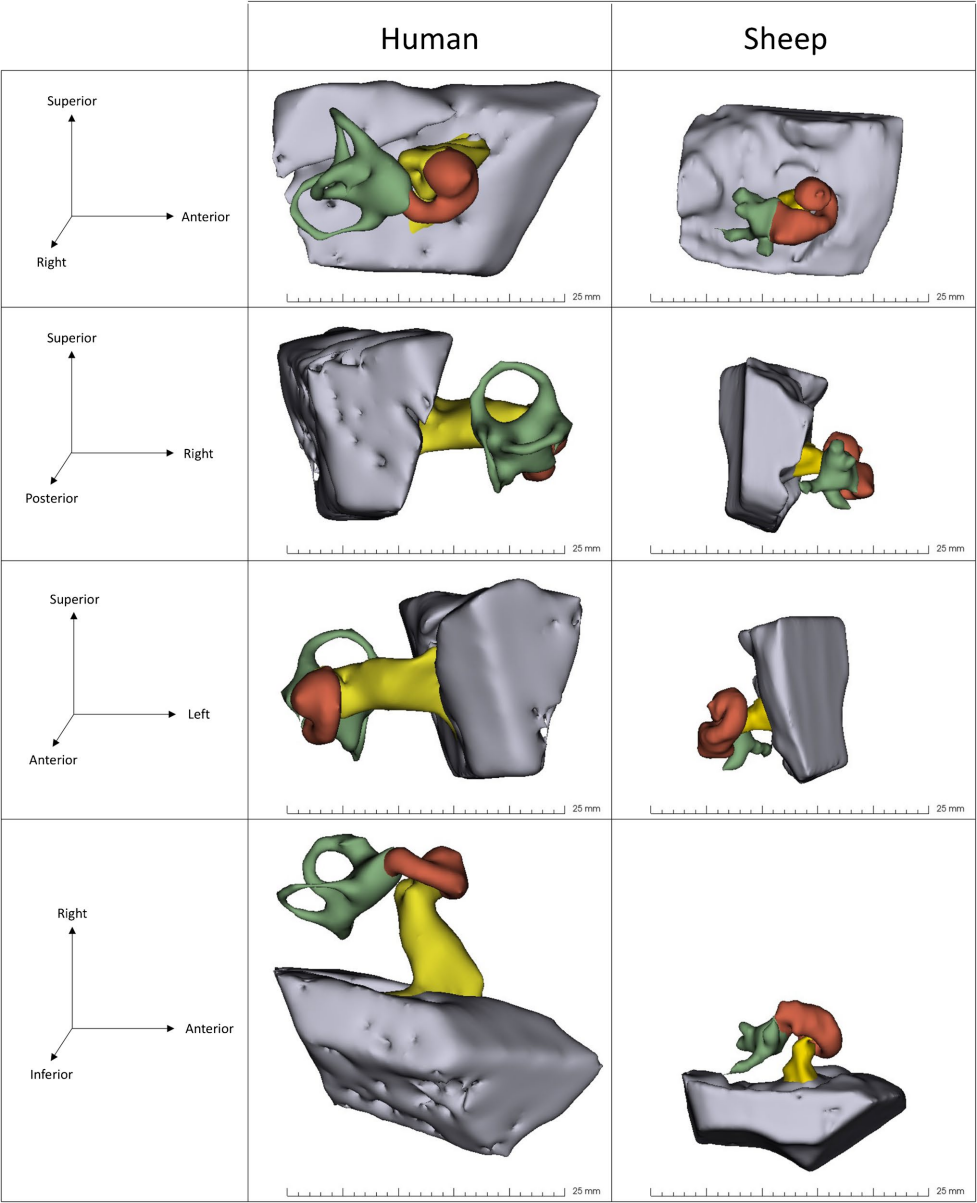
Comparison in 3D representation using 3D Slicer^®^ software of the sheep and human right IE at the same scale in different planes of space. The red part corresponds to the cochlea (anterior labyrinth), green to the vestibule (posterior labyrinth) and yellow to the internal auditory canal. The grey area corresponds to the posterior cerebral fossa. Given the resolution of the MRI, the semicircular canals were not fully visualized in the sheep. The difference in length of the internal auditory canal between both species can be observed

The radiological anatomy of both right and left IEs of sheep and humans were analyzed and compared (Fig. 4). No anatomical cochlea-vestibular malformation was observed in the MRI scans of the sheep and human IEs.

In all sheep, a wave IV was detected at 20 dB HL or 30 dB HL, confirming normal hearing function. The MRI scans were compared between sheep and humans. IE morphologic study showed the presence of the three semi-circular canals, the vestibule, and the cochlea for all humans. In sheep, the MRI intensity thresholds were reduced due to the limited visibility of the semicircular canals in the IE and the L_total_ could not be measured. The mean L_total_ was 17.53 ± 0.70 mm in humans. All measurements were significantly larger for human than for sheep IEs (*p* = 0.0002 for each measurement), except for the number of spiral turns of the cochlea (*p* = 0.37) (Tables 1 and 2). There was no significant difference in cochlea volumes between the left and right IEs in either the human (*p* = 0.485) or sheep models (*p* = 0.685). Sheep and human were compared in terms of ratios. All ratios were approximately 2/3 between sheep and humans (mean ratio 0.66, range between 0.59 and 0.72) except for the IAC_L_ with a ratio of 0.31 (approximately 1/3) and the number of spiral turns of the cochlea (ratio = 0.94, with 2.33 spiral turns for humans and 2.25 turns for sheep).

**Table 1:** Sheep inner ear radiological measurements from MRI scans

Sheep	Side	C_L_ (mm)	C_W_ (mm)	Cochlea volume (mm^3^)	IAC_L_ (mm)	IAC_W_ (mm)	Scala tympani length (mm)	Spiral turns of the cochlea (number)
1	R	6.47	4.25	45.93	3.85	2.17	20.29	2
2	L	7.10	4.20	47.78	3.29	2.66	20.72	2
3	R	7.45	4.10	53.64	3.84	1.92	23.04	2 ½
4	L	7.21	4.21	55.55	3.90	2.36	22.84	2 ½
5	R	6.41	4.26	57.53	3.80	2.04	22.31	2 ½
6	L	6.76	4.20	67.34	3.90	2.19	21.71	2 ¼
7	R	7.13	4.55	62.30	4.16	1.91	19.67	2 ¼
8	L	7.67	4.45	61.54	4.20	1.89	21.35	2
Mean SD		7.03	4.28	56.46	3.87	2.14	21.49	2 ¼
		0.44	0.14	7.31	0.27	0.26	1.21	0 ¼
Sheep/human size ratio		0.76	0.66	0.62	0.31	0.59	0.68	0.96

*C*_*L*_: cochlea length; *C*_*W*_: cochlea width; *IAC*_*L*_ length of the inner auditory canal; *IAC*_*W*_: width of the inner auditory canal

**Table 2:** Human inner ear radiological measurements on MRI-scan

Humans	Side	C_L_ (mm)	C_W_ (mm)	Cochlea volume (mm^3^)	IAC_L_ (mm)	IAC_W_ (mm)	Scala tympani length (mm)	Spiral turns of the cochlea (number)
1	R	8.96	6.20	85.91	11.73	3.87	33.21	2 ½
2	L	9.07	6.00	87.86	11.53	3.96	29.53	2 ¼
3	R	9.72	6.35	94.17	13.47	3.50	28.76	2
4	L	9.81	6.60	97.46	14.32	4.21	29.09	2 ¼
5	R	9.07	6.53	90.61	12.7	3.89	33.28	2 ½
6	L	9.38	6.51	95.23	12.35	3.97	32.23	2 ½
7	R	8.66	6.61	85.07	10.72	2.77	31.87	2 ½
8	L	8.74	6.44	85.19	12.46	2.81	32.91	2 ½
Mean SD		9.18	6.41	90.19	12.41	3.62	31.36	2 ${~}^{1}\!\left/ \!{~}_{3}\right.$
		0.42	0.21	4.91	1.13	0.54	1.91	0 ${~}^{1}\!\left/ \!{~}_{10}\right.$

*C*_*L*_: cochlea length; *C*_*W*_: cochlea width; *IAC*_*L*_ length of the inner auditory canal; *IAC*_*W*_: width of the inner auditory canal

## Discussion

This study supported that the sheep cochlea was 2/3 the size of the human cochlea across all measurements, except for the internal auditory canal, where a ratio of 1/3 was observed. The number of spiral turns in the cochlea was identical in both species.

Prior to conducting research on humans, large animal studies are crucial, and this is especially true when investigating small anatomical systems. To the best of our knowledge, there are no previous studies that compare the volume of the IE between large animals and humans. High-resolution MRI proved to be a powerful modality for visualizing structures of the IE and comparing volume and dimensions between humans and sheep, enabling detailed quantitative analysis of these complex anatomical features. In the present study, the human cochlea had an average volume of 90.19 mm^3^, a length of 9.18 mm, and a width of 6.41 mm. These measurements correspond closely to those reported in the literature – 94.42 mm^3^ in volume [12], 9.2 mm in length, and 7 mm in width [13]. This study is the first to examine the sheep’s IE using MRI, revealing a size ratio of approximately 2/3 between sheep and humans. These findings are consistent with the previously described literature (Table 3).

**Table 3:** Dimensions of the sheep's inner ear described in the literature

Studies	Sample size	Sheep breed	C_L_ (mm)	C_W_ (mm)	Cochlea volume (mm^3^)	IAC_L_ (mm)	IAC_W_ (mm)	Scala tympani length (mm)	Spiral turns of the cochlea (number)
Trinh et al*.* [9]	10	Ile de France	7.67	5.18	48.1	ND	ND	24.37	2 ½
Schnabl et al*.* [15]	3	ND	ND	ND	ND	ND	ND	34.1	2 ¼
Seibel et al*.* [16]	19	Corriedale and Texel	8.20	ND	ND	2.00	1.60	19.9	ND
Soares et al*.* [17]	8	Corriedale	ND	ND	ND	ND	ND	ND	2 ½
Present study	4	Ile de France	7.03	4.28	56.46	3.87	2.14	21.49	2 ¼

*C*_*L*_: cochlea length; *C*_*W*_: cochlea width; *IAC*_*L*_ length of the inner auditory canal; *IAC*_*W*_: width of the inner auditory canal; *ND*: No data

For several years, otologists have shown interest in the sheep model, both for research and surgical training purposes [14]. While many research groups have focused on describing the anatomy of the sheep's middle ear [14–22], fewer have studied the IE anatomy [9, 14, 16, 17]. In the present study, the ratio of scala tympani length between sheep and humans was 0.68 (21.49 mm in sheep and 31.36 mm in humans). Histological studies in humans have measured a scala tympani length of 36 mm [6], which is greater than the value found in the present study. This discrepancy may be due to MRI resolution limitations, which make it challenging to visualize the cochlear apex. Beyond 29 mm in humans, the height of the scala tympani is less than 0.4 mm, matching the resolution threshold of MRI slices [6]. The resolution may also account for the slightly lower number of cochlear turns in humans (2.33 turns) in the present study, compared to the average 2.6 turns reported in the literature, with a range of 2.2–2.9 turns (described using method of casting temporal bone specimens) [23]. Additionally, differences in sheep strains may contribute to the variation seen across studies [21].

A significant difference was found in the IAC_L_, with a ratio of 1/3 between sheep and humans. Only one previous study has evaluated the IAC_L_ in sheep using a CT-scan, reporting a mean of 2.00 mm and a ratio of 1/6 compared to humans [16]. This previous study identified an average human IAC_L_ of 13.0 mm, which closely aligns with the 12.41 mm observed in the present study [16]. Although young sheep were used in this study (mean age = 3.57 years, with a life expectancy of approximately 14 years [24]), sheep are fully mature by 24 months [24]. Therefore, it is unlikely that age influenced the smaller IAC_L_ measurements in sheep in the present study. This is supported by research showing that human IAC_L_ differs by only 0.2 mm between adolescence and adulthood [25].

Surgical dissections are essential for honing surgical skills because they help develop dexterity and improve outcomes in future procedures by minimizing errors. While cadaveric dissections are the gold standard for acquiring surgical proficiency, access to cadavers is limited in some countries due to high costs and strict regulations, creating challenges for training young otologists. In contrast, animal models offer a cost-effective, reliable alternative, with the ovine model proving particularly suitable. Anschuetz et al*.* developed an ex vivo endoscopic surgical training atlas using sheep [26]. Various procedures, including myringoplasty [27, 28], ossiculoplasty [26, 27], and stapedectomy [19, 29] can be practiced on this sheep model. Although sheep have a poorly pneumatized mastoid [15, 17, 19], the approach to the facial recess via mastoidectomy has been well documented [21], and cochlear implantation, using human electrode array, has been successfully performed on this model both ex vivo [9, 15, 30] and in vivo [31]. Overall, sheep represent a relevant surgical training model.

Given the anatomical and physiological similarities, sheep serve as an excellent model for auditory research. Some research groups are focusing on developing minimally invasive robotic cochlear implant devices to address hearing loss [32]. Others are exploring methods in sheep to restore hearing by improving the local delivery of therapeutic agents to the IE, such as hydrogels [33] or gas microbubble-assisted ultrasound [34]. While transgenic models in large animals are costly and more complex to develop than in small animals [11], transgenic sheep models are now available [35], although none have been specifically designed for IE pathologies. Beyond devices and procedures, the volume of IE fluids is a critical factor when comparing studies involving perilymph sampling for pharmacokinetic analysis, biomarker research, or drug delivery [11]. In this study, the contrast between the MRI signal intensity of the perilymph and endolymph was more pronounced in sheep, possibly indicating a difference in endolymphatic fluid composition between sheep and humans. Limited data exists in the literature comparing perilymph in humans and other species. Some studies have compared perilymph protein compositions, revealing a 59% similarity between humans and mice [36] and 64% between humans and guinea pigs [37]. Metabolomic studies have also been conducted [38], although these rely on human metabolomic databases [39], and no ovine-specific metabolomics database currently exists. Thus, extrapolating metabolomic findings from animal models to humans remains challenging.

Other large animal models can be considered for research purposes, but they present certain disadvantages. As for example, pigs have a thicker layer of soft and fatty tissues in the mastoid region, complicating the surgical approach for cochlear implantation [15]. Additionally, their weight, often around 300 kg, makes them more difficult to handle compared to sheep, which typically weigh around 70 kg [11]. Primates, such as macaques, offer a closer phylogenetic and anatomical resemblance to humans [40], but their use is highly restricted due to ethical regulations [30].

There were a few limitations to the present study. The same MRI parameters were used for both humans and sheep, with 0.4 mm thick slices. Given that the sheep's IE is approximately 2/3 the size of the human IE, these slices were insufficient to fully visualize the semicircular canals, making it impossible to measure the volume and length of the vestibule. To capture the semicircular canals in their entirety, 0.3 mm sections would be more suitable. This would also enable more accurate measurements of the cochlear turns and the length of the scala tympani. The development of 7 T MRI for humans and animals could significantly enhance the resolution of MRI acquisitions, thereby allowing for the distinct visualization of the various endocochlear compartments [41].

## Conclusion

The present morpho-anatomical study of the sheep cochlea suggests that the sheep is a promising model for IE research, particularly for investigating cochlear function and pathologies. Indeed, the sheep cochlea shows dimensions that were proportionally similar to that of humans. The near-equivalent number of cochlear turns further supports the use of sheep as a relevant model. However, some variations, such as the smaller size of the internal auditory canal, should be considered in future research.

## Supplementary Information

Below is the link to the electronic supplementary material.

Supplementary file1 Video showing MRI sections passing through the inner ears of sheep (right and left) represented in the different planes (left: axial plane, top right: coronal plane, bottom right: sagittal plane). (MP4 18831 KB)

## Data Availability

Not Applicable.
